# Bilateral viral keratitis following corneal collagen crosslinking for progressive keratoconus

**DOI:** 10.1186/s12348-019-0185-8

**Published:** 2019-08-28

**Authors:** Sanjeeta Sitaula, Sanjay K. Singh, Anil Gurung

**Affiliations:** 1 B.P.Koirala Lions Centre for Ophthalmic Studies, Maharajgunj Medical Campus, Institute Of Medicine, Kathmandu, 44600 Nepal; 2 Department of Cornea Clinic, Biratnagar Eye Hospital, Biratnagar, Nepal; 3 Geta Eye Hospital, Kailali, Nepal

**Keywords:** Corneal collagen crosslinking, Herpetic keratitis, Keratoconus

## Abstract

**Purpose:**

Corneal collagen crosslinking has been proven to be a useful technique to slow the progression of keratoconus. With its increasing use, we are encountering rare complications. We describe a case that developed bilateral viral keratitis after corneal collagen crosslinking with riboflavin and ultraviolet A for progressive keratoconus.

**Case report:**

An 18-year-old boy underwent corneal collagen crosslinking in both the eyes at the same setting for bilateral progressive keratoconus. He was discharged with a soft bandage contact lens and asked to follow up in 5 days. Seven days later, the patient returned with severe pain, redness, and photophobia for the last 2 days. The bandage contact lens was removed. There was a central corneal lesion in a branching dendritic pattern in both the eyes and the corneal sensation was reduced. Based on the findings, a clinical diagnosis of bilateral viral keratitis was made. The dendrite healed completely in 10 days with oral and topical acyclovir treatment, and the cornea had a faint scar at 1 month follow-up with best-corrected visual acuity of 6/9 in both eyes with a rigid gas permeable lens.

**Discussion and conclusion:**

Ultraviolet A light could be a stimulus to trigger reactivation of latent HSV infections even in patients with no history of clinically evident herpes virus ocular infections. Early diagnosis and timely treatment can have good visual outcome. Prophylactic antiviral medication may be useful to prevent this complication in individuals with prior history of viral keratitis.

## Introduction

Keratoconus is noninflammatory degenerative and progressive corneal ectasia of the cornea, resulting in corneal thinning, progressive myopia, and irregular astigmatism. Various treatment modalities currently available for treatment of KC include rigid gas permeable lenses, penetrating keratoplasty, deep anterior lamellar keratoplasty, or intrastromal corneal ring segments which can only correct the refractive errors of keratoconus but cannot treat the underlying cause. The technique of corneal collagen crosslinking (CXL) was described first in human subjects as a useful method to slow or even halt the progression of keratoconus by Wollensak et al. [1] and many other studies have shown similar beneficial effect with CXL [2–5]. Although crosslinking is a low-invasive procedure with a very low rate of complications secondary keratitis is a potentially sight-threatening complication that can occur. Here, we describe a case report of a patient who developed bilateral viral keratitis after corneal collagen crosslinking. Written informed consent was provided by the patient for publication of the case details and images.

## Case report

An 18-year-old boy was referred to our center for bilateral progressive keratoconus. He gave history of blurring of vision for distance with rapid change in the power of glasses in the past year. He had occasional itching. On examination, unaided distance visual acuity was 3/60 and 6/60 in his right eye (RE) and left eye (LE) respectively. On retinoscopy, there was scissors reflex seen in both the eyes. Retinoscopic findings in the RE was − 8.00D/− 7.00Dc × 20° and in the LE was − 4.5D/− 6.00Dc × 160°. On examination of the cornea, there were Vogt’s striae and Fleischer’s ring in both eyes and Munson’s sign was positive in LE. The corneal topography showed bilateral central steepening with features suggestive of keratoconus.

In the view of the progressive nature of keratoconus, corneal collagen crosslinking was done in both the eyes at the same setting in a sterile environment of the operating room using the Dresden protocol. Proparacaine 0.5% was used topically for anesthesia. Lid speculum was applied. Central 8 mm of corneal epithelium was debrided using ethanol application on the cornea using cotton-tipped buds on both eyes (BE) for 20 s. Riboflavin sodium phosphate 1 mg in 20% dextran was instilled every 3 min for 30 min prior to the procedure in BE. Ultraviolet A (UVA) irradiation (365 ± 5nmSD OptoXlink 1.0 cross linking device) via 9 mm aperture at 45 mm distance from the apex of cornea was used first in RE and then LE for 30 min in each eye. The parameters used were time 30 min, dose 5.405 J/c, power 1.51 mW, and intensity 3.003 mW/cm^2^. During irradiation, riboflavin was continuously instilled at every 5-min interval. At the end of the procedure, ofloxacin eye drops were applied and bandage contact lens were applied in BE.

**Fig. 1:**
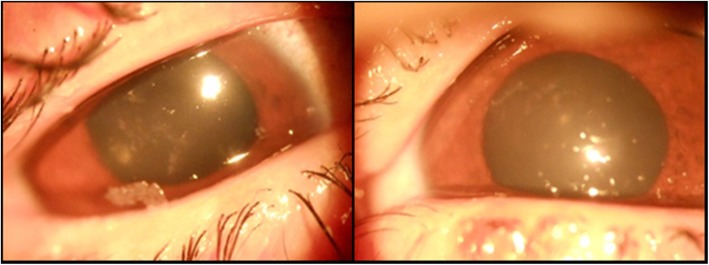
Right and left eye showing central corneal lesion in a dendritic pattern

**Fig. 2:**
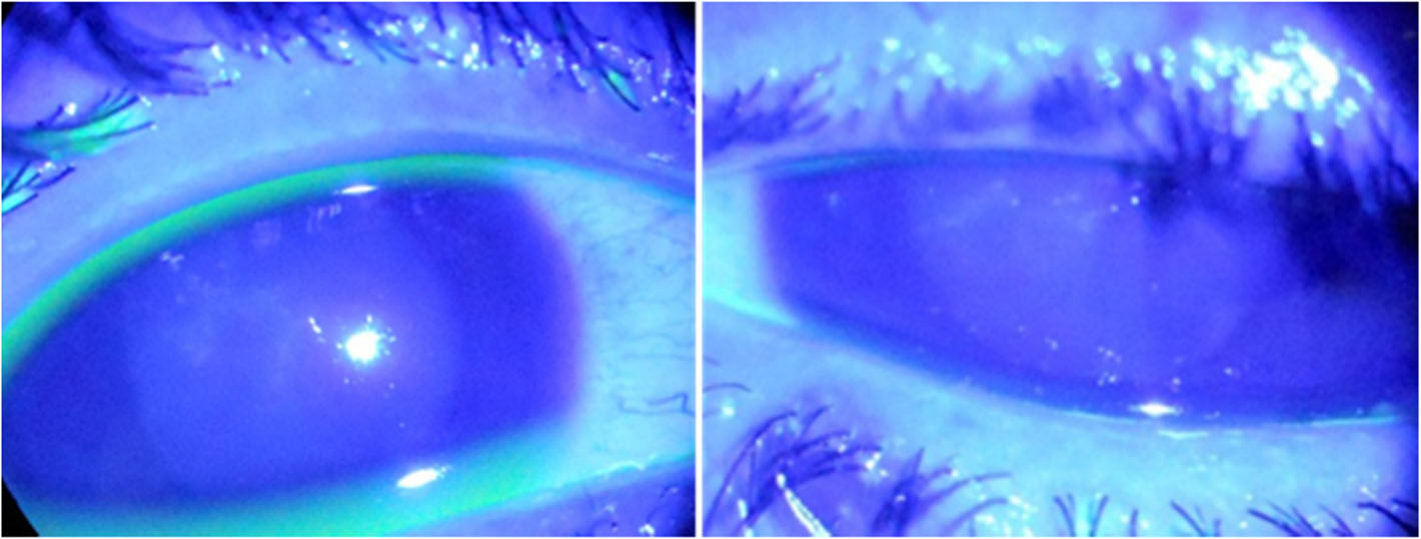
Right and left eye showing central corneal lesion in a dendritic pattern with minimal fluorescein uptake in cobalt blue filter

The patient was discharged the following day with ofloxacin 0.3% drops six times, carboxymethyl cellulose drops six times, and flurometholone 0.1% eye drops two times a day and asked to follow-up after 5 days. The patient returned after 7 days complaining of pain and redness for the last 2 days. The bandage contact lens was removed. On examination, there was circumciliary congestion with central corneal infiltrate in a branching dendritic pattern in both the eyes (Figs. 1 and 2). The corneal sensation was decreased in both the eyes. The anterior chamber was quiet.

The patient did not have any history past redness, atopy, or any viral infection in any of his eyes prior to this episode. He was immunocompetent and did not have any underlying systemic disease. We did not have the facility for polymerase chain reaction for confirmation of the diagnosis of herpes infection. Thinking in terms of viral keratitis topical flurometholone was stopped and oral acyclovir 400 mg five times was started along with ointment acyclovir 3% five times, ofloxacin eye drops four times, and carboxymethylcellulose eye drops four times was started.

The patient improved symptomatically with treatment and the dendrite healed completely within the next 10 days leaving behind superficial punctate keratitis. Oral acyclovir was stopped and topical acyclovir was tapered and stopped in a month. The cornea was clear at 1 month with BCVA of 6/9 in BE corrected with rigid gas permeable lens.

## Discussion

In this case report, we describe a patient who developed bilateral herpetic keratitis after CXL. Fortunately, the dendrite healed completely with antiviral treatment and the cornea was clear at 1 month follow-up. Corneal collagen crosslinking is finding its use in a number of corneal ectatic diseases because of its effect in increasing the biomechanical strength of the cornea. Among the various complications of this procedure, reactivation of viral keratitis or the occurrence of fresh viral keratitis has been reported in other case reports in literature [6–9]. Herpetic Eye Study Group did not find any significant association between recurrence of herpetic keratitis and factors like psychological stress, systemic infection, sunlight exposure, menstrual cycle, and contact lens wear [10]. However, other studies have reported reactivation of herpes simplex virus (HSV) after emotional stress, corneal surgery, trauma, fever, exposure to UV radiation, and after laser surgery [9, 11–13]. Our case report and all the four other cases [6–8] reported did not have a prior history of clinical herpetic ocular infection, so it may be that UVA light could be a stimulus to trigger reactivation of latent HSV infections even in patients with no history of clinically evident herpes virus ocular infections. The exact mechanism of herpes reactivation still remains unknown. Factors like epithelial trauma or damage of the corneal nerves could be the mechanism of HSV reactivation. Other possible factors could be the use of topical corticosteroids and mechanical trauma caused by epithelial debridement which was a common factor in our case and all the other cases after CXL [6–8]. Prophylactic systemic antiviral treatment in patients with a history of herpetic disease after crosslinking with UVA might decrease the possibility of recurrence

Herpetic keratitis is usually unilateral. Bilateral involvement is seen more often in young individuals, patients with a history of atopy, or patients with immune deviations. Our case was also a young man although he did not have atopy or any immunocompromised condition. The use of topical steroid and the procedure itself may have predisposed bilateral herpetic keratitis in our patient. Performing crosslinking in both the eyes at the same setting requires a proper counseling and a cooperative patient as the patient has to sit still in a lying position for almost 90 min. We performed crosslinking in both the eyes in the same setting in our patient as he was from a distant place and had difficulty in follow-up as he was appearing for his board exams that year.

All the other cases report unilateral herpetic keratitis as the CXL was done in only one eye at a time. Ours is the only case reported in literature where CXL was done in both the eyes at the same sitting and bilateral herpetic keratitis occurred. Although we were not able to confirm the diagnosis by laboratory studies, our diagnosis of bilateral herpetic keratitis was supported by the chain of events like the pain that had initially subsided was suddenly exacerbated with the appearance of dendritic lesions in both the central cornea after the epithelial defect had almost healed. The corneal sensations were also markedly decreased in both the corneas and the lesions responded very well to oral and topical acyclovir treatment.

As crosslinking is emerging as a standard treatment for keratoconus, various complications of crosslinking are also becoming more evident, especially with the epithelial off technique out of which one is infective keratitis. Corneal collagen crosslinking (CXL) has been studied as an alternative option in the management of corneal infections by its effect of blocking corneal melting and its antimicrobial properties termed as “PACK-CXL”: Photo Activated Chromophore for Keratitis Crosslinking [14]. However, CXL done for viral keratitis led to deterioration of ulcer and melting in various reports [15, 16]. Hence, viral reactivation following CXL with UVA is a potential complication, and prophylactic oral antivirals may be beneficial in a patient with prior history of herpes infection. Similarly, avoiding bilateral simultaneous treatment in all patients is preferable.

## Data Availability

Not applicable

## Abbreviations

BE: Both eyes
CXL: Corneal collagen crosslinking
HSV: Herpes simplex virus
LE: Left eye
RE: Right eye
UVA: Ultraviolet A
